# An update on the strategies in multicomponent activity monitoring within the phytopharmaceutical field

**DOI:** 10.1186/1472-6882-12-18

**Published:** 2012-03-14

**Authors:** Johanna M Gostner, Oliver A Wrulich, Marcel Jenny, Dietmar Fuchs, Florian Ueberall

**Affiliations:** 1 Division of Medical Biochemistry, Biocenter, Innsbruck Medical University, Fritz-Pregl-Str. 3, 6020 Innsbruck, Austria; 2 Division of Biological Chemistry, Biocenter, Innsbruck Medical University, Fritz-Pregl-Str. 3, 6020 Innsbruck, Austria

**Keywords:** Multicomponent, Multitarget, Effect potentialization, Microarray, Network

## Abstract

**Background:**

To-date modern drug research has focused on the discovery and synthesis of single active substances. However, multicomponent preparations are gaining increasing importance in the phytopharmaceutical field by demonstrating beneficial properties with respect to efficacy and toxicity.

**Discussion:**

In contrast to single drug combinations, a botanical multicomponent therapeutic possesses a complex repertoire of chemicals that belong to a variety of substance classes. This may explain the frequently observed pleiotropic bioactivity spectra of these compounds, which may also suggest that they possess novel therapeutic opportunities. Interestingly, considerable bioactivity properties are exhibited not only by remedies that contain high doses of phytochemicals with prominent pharmaceutical efficacy, but also preparations that lack a sole active principle component. Despite that each individual substance within these multicomponents has a low molar fraction, the therapeutic activity of these substances is established via a potentialization of their effects through combined and simultaneous attacks on multiple molecular targets. Although beneficial properties may emerge from such a broad range of perturbations on cellular machinery, validation and/or prediction of their activity profiles is accompanied with a variety of difficulties in generic risk-benefit assessments. Thus, it is recommended that a comprehensive strategy is implemented to cover the entirety of multicomponent-multitarget effects, so as to address the limitations of conventional approaches.

**Summary:**

An integration of standard toxicological methods with selected pathway-focused bioassays and unbiased data acquisition strategies (such as gene expression analysis) would be advantageous in building an interaction network model to consider all of the effects, whether they were intended or adverse reactions.

## Background

Prior to the 20th century, medicine relied almost exclusively on the use of natural products or botanically-derived multicomponent therapeutics. Today, at least 25% of all pharmaceuticals are based on plant-derived products. However, although the very earliest pharmaceutical products were botanical and/or natural multicomponent preparations, by now mainly purely isolated monocompounds or synthetic analogues are commercialized as conventional drugs [1,2]. The development of pharmaceutical and chemical technologies facilitated the economical production of semi-and fully-synthetic monocompound drugs, saving resources, including time, labor and delivery costs. Furthermore, characterization, standardization, and quality control of active ingredients became less difficult due to the absence of assay-interfering compounds in complex mixtures [3].

The shift towards the favoured use of monocompound drugs was supported by the finding that, in some plants, single components were the basis for efficacy. The isolation of these active substances enhanced their therapeutic effectiveness and allowed for dose assessments. For some time, the major demand of the pharmaceutical industry has become the discovery of a new drug entity that interacts with a single, well-defined molecular target, preferably without disturbing other cellular functions to avoid side effects [4]. Prominent druggable targets are, for instance, key molecules that are responsible for disease development and/or progression. Thus, drugs binding to such proteins should, in theory, lead to alterations or inhibition of their activities.

Hence, a general limitation of *in silico *drug-target interaction and activity modelling procedures is their inability to mimic entire cellular processes. Despite the use of sophisticated design strategies for selective drug ligands, in analogy to the "lock and key" concept, only a few of these monocompounds have been proven to be successful *in vivo *[5]. Interestingly, Roth, Hopkins and colleagues proposed that many modern anti-psychotic drugs failed in the clinic because they were too selective for their specific targets [4,6]. Furthermore, an analysis of approved drugs indicates that the modulation of several molecular targets is a frequent mechanism behind drug efficacy [4].

An additional disadvantage of single drug therapies is the development of resistance phenomena, which may occur on a biochemical level, be acquired, and/or be established on a genetic level. Multidrug therapy has become especially important in the fight against infectious diseases. Several approaches used to evaluate the activities of antimicrobial drug and natural product combinations have been reviewed extensively [7]. Additionally, there have been reports on the reduction in the occurrence of resistance of antimicrobial strains to crude rather than single active compounds, e.g. from antimalarial drug research [7,8].

Often, the aetiology of diseases that involve a polygenic background and environmental factors remains poorly understood. Consequently, this complicates the selection of proper drug targets in drug design. Additionally, it results in the simultaneous use of multiple drugs for the treatment of disease symptoms rather than origins, involving therapeutics that have not been developed or analyzed with respect to drug-drug interactions. The increasing demand for polypharmacology to enhance treatment efficacy via multitarget interventions is an attempt reflected e.g. by the search for synergistic combinations of single drugs, or by the selective design of non-selective, multi-target directed drugs or molecular entities containing two functionally distinct pharmacophores [4,6,9].

A strong interest in multicomponent phyto- or natural product preparations is likely to arise from the observation that some of these multi-substance mixtures possess prominent pharmacological properties at low or non-toxic concentrations. However, due to their complex chemical composition, an understanding of the underlying molecular activity mechanisms is, in most cases, only superficial. Thus, a detailed 'mechanisms of action' analysis is urgently required to unravel the potent activity of phytochemicals and botanical remedies, as well as to predict potential drug-drug interactions that may result from multidrug treatments.

A glossary of terms frequently used is provided in Table 1.

**Table 1:** Glossary

Active marker	Constituents of pharmaceutical relevance, which contribute to or influence the activity of extracts [10].
**Active substance/active pharmaceutical ingredient**	Substances that exert a biological activity, which provoke a specific effect in a biological system. Active principles can exhibit activities comparable to that of synthetic active substances [10].

**Analytical marker**	Chemically-defined substances used for quality control and standardization procedures, selected according to their analytical value without dependence on potential therapeutic activities [10].

**Botanical**	One or more plants, a plant part, or an extract valued for its nutritional, medicinal, or therapeutic properties. Herbs are a subset of botanicals [11].

**Botanical drug**	Highly, but not completely characterized, complex extracts from plants that are clinically evaluated for safety and efficacy. Often, there is also a history of safe traditional human use [2].

**Dietary supplement/functional food**	Consist of components that are supplemented in the diet or thought to be healthy, such as vitamins, minerals, fats, botanicals, etc. [12].

**Interaction/joint action**	Actions that describe an altered outcome arising from the presence of two or more compounds that could be **antagonistic, additive, or synergistic **[13]. **Potentiation **results from additive and synergistic interactions that intensify the potency of a bioactive product [2,14].

**Multicomponent/complex interventions**	Either a mixing of pharmaceuticals, an intended administration of a multicomponent combination of reference listed drugs, or the uptake of multicomponent preparations (e.g. botanicals, natural products or dietary supplements). Keith *et al. *defined multicomponent drugs as a therapeutic regimen that consists of a concerted pharmacological intervention of several compounds that interact with multiple targets [15].

**Natural product**	All substances produced by living organisms, including microorganisms or fungi.

**Nutraceutical(s)**	Provide medical or health benefits, including the prevention and/or treatment of a disease, in addition to preventing nutritional deficiencies [12].

**Nutrigenomics**	Studies the interaction between dietary components and/or nutrients and the genome by focusing on changes in transcript, protein and metabolite levels [16,17].

**Pharmacogenomics**	Combines conventional pharmaceutical and toxicology study designs with global genomics technologies and appropriate disease model systems to provide a comprehensive view on the response of the genome and the biochemical machinery of the cell upon treatment, and to identify efficacy and toxicity-related mechanisms [18,19].

**Phytochemical(s)**	Non-nutrient biologically active compounds in plants. A number of databases that e.g. contain information on chemical structure, metabolic pathways or health-related properties in humans have been only recently reviewed by Scalbert *et al. *[20].

**Secondary metabolite**	Substances produced by plants or microorganisms that are not necessary for primary or energy metabolism, but are important for ecological fitness. Evolutionary pressure on biosynthetic pathways resulted in an inexhaustible chemical diversity of substances, and some of them have potent pharmaceutical properties [21].

### The mechanism(s) of phytochemical action can either be direct or indirect

Most plant-derived bioactive substances are secondary metabolites. These compounds are produced as chemical signals in response to environmental changes or as defence mechanisms against pathogens, herbivores, and environmental stress factors. The most important structural classes of secondary metabolites are nitrogen-containing alkaloids, terpenoids, steroids, and phenolics (mostly phenylpropanoids) [3]. Although a few of these phytochemicals are now established as potent monosubstance drugs in modern medicine, most of them have not been structurally characterized and/or explored regarding their potential beneficial effects for human health.

In general, most monosubstance phytodrugs exhibit a rather specific mechanism of action. For instance, the cardiac glycosides from *Digitalis *spp. are able to inhibit membrane Na^+^/K^+ ^ATPases. Conversely, according to Wink, the pharmacological activity of multicomponent mixtures cannot be assigned to a single substance, and the contained phytochemicals typically act in an unspecific and widespread manner [22]. Prominent bioactive components might be part of the preparation, but in some cases, there are no apparent single active components, which are detectable and responsible for the net effect. Interestingly, synthetically-developed multi-target drugs are also sometimes low affinity binders, since the multitude of low-affinity and/or transient interactions is sufficient to achieve a significant modification [23].

Multicomponent activity spectra result from the combined and simultaneous attack on various central cellular target structures. Examples of such non-specific targets are biomembranes, gene regulatory elements, and proteins. Proteins can be affected either by introducing covalent bindings, interfering through weak, but multiple non-covalent interactions, or the deposition of lipophilic compounds in hydrophobic regions of proteins e.g. within substrate binding pockets, which may cause loss of activity, total inhibition, or degradation. Furthermore, changes in the activity of signalling molecules or transcription factors can lead to an induction of transcriptional responses. Lipophilic and amphiphilic substances are poorly soluble in the cytosolic compartment, and have the tendency to accumulate spontaneously on biomembranes, where they can influence membrane fluidity and density. If nucleic acids are targeted, mutations can be initiated by the introduction of covalent modifications or through intercalation mechanisms [22].

Thus, despite a very low molar fraction and weak impact of individual phytochemicals in multicomponent mixtures, the summation of activities leads to a potentiation of effects, and promotes a prominent outcome. This mechanistic view also explains why in many cases, the fractionation or isolation of principal constituents from extracts ends up with a loss of previously detected activities [15].

### Different types of joint actions contribute to the potentiation of effects

Compounds exert their bioactivities by interacting with other molecules rather than by acting alone. Numerous theories propose that the interplay and interference of single components in a mixture is the rationale for the advantageous effects of multicomponents. The basic concepts of joint actions and interactions can be summarized as three core processes: addition, synergism, and antagonism [13,14,24-26].

Additive interactions are based on either similar or dissimilar non-interactive effects of chemicals. The net response of a multicomponent may be attributed to the sum of the individual compound doses or effects. Similar actions preferentially occur with structurally-related substances, while dissimilar acting chemicals differ in their mechanism of action, but share the resulting effect [13]. In contrast to independent actions, synergistic and antagonistic processes require direct interactions. These interactions lead to an effect that is stronger, as expected based on the dose or response of each component. Antagonistic interactions result in an inhibition of effects, while synergistic interactions lead to effect potentiation. In both cases, the net effect exceeds the additive/subtractive effects of each component [13,14]. Thus, using synergistically-acting compounds lowers the amount of potentially harmful chemicals necessary to achieve an optimal therapeutic efficacy. Furthermore, interactions between components within a single plant species (endointeractions), and interactions between components from different plants and/or non-plant based compounds (e.g. synthetic drugs), which may be ingested together (exointeractions), can be distinguished [27].

The concepts of joint actions are helpful in the understanding of basic processes. The effectiveness of joint actions may result from the local and spatial proximity of components at the target site of action, which only occurs if the substances possess similar absorption and circulation kinetics [13,28]. Additionally, substances that interfere with general cellular mechanisms, such as oxidative stress or protein folding, can further interact in a time-independent manner with compounds that act on target molecules belonging to different pathways.

However, the response to substances of a highly interconnected biological system, such as the human organism, is very complex and rarely linear. There are a variety of factors that can influence the therapeutic efficacy of a substance, such as the effective intracellular concentration of compounds at the target cell or organ (e.g. bioavailability, bioconversion, pharmacokinetics), the chemical and physical microenvironment (e.g. polarity, viscosity) at the interaction site, the type of molecular target (e.g. single or multiple molecules, cellular structures), and the general health of the target cells.

### Multicomponent interventions for multifactorial diseases

Many of today's illnesses are thought to result from environmental and lifestyle changes, which favour an undesirable functioning of biological systems that have evolved over an evolutionary time course [29]. These diseases are caused by multiple factors, unfold over an extended period of time, and demonstrate a wide range of pathophysiological manifestations.

One such example is atherosclerosis, a chronic inflammatory disease. Initial atherosclerotic events take place long before its clinical manifestations within the vascular system can be diagnosed [30,31]. According to Ramsey *et al*, a more systems-based view on multifactorial disorders, where disease progression requires the coordination of several cell types, organs, and organ systems at various molecular levels, will contribute to a better understanding of disease aetiology [32]. Of note, treatment options for atherosclerosis-associated diseases, which are discussed extensively in scientific literature, also include the use of herbal extracts and mixtures that contain, among others, phenolic antioxidants, which serve as protective agents [33].

Also, several psychiatric and neurologic disorders are the result of multifactorial interactions between environmental influences and genetic mechanisms. A variety of modern central nervous system targeting monocompound drugs have been originally identified in psychoactive plants and natural products, which are components of many traditional medicinal systems [34]. As many traditional medicinal formulations are based on the combined and synergistic activities of several substances that enhance their pharmacological properties, investigations of the resulting mechanisms of action could help to uncover new medications for the treatment of diseases of the nervous system and mental illnesses. Furthermore, other factors, such as the general state of health or diet, should be considered [35]. For example, disorders accompanied with cellular immune activation such as infections, or persisting deficits in nutrition, may diminish serum tryptophan levels, which consequently results in the reduced production of serotonin and may affect serotonergic functions [35,36].

Due to the broad range of activities possessed by multicomponents, there may be numerous promising candidate mixtures that achieve benefits in the treatment of such multifactorial diseases likely by shifting, rather than interrupting, cellular regulation towards a healthy level [37,38]. The regulatory cellular and molecular mechanisms in an organism have to be organized by high interconnectivity to fulfil complex functions, such as signal sensing, transduction and processing, and thereby maintain systemic and cellular homeostasis. The sophisticated organization of intra-and intercellular communication facilitates an appropriate and flexible response to disturbances and provides control mechanisms through interlocking pathways, as well as feedback, regulatory, and fail-safe mechanisms [29]. While initial quick signalling is mediated generally through specialized sensory molecules or complexes, it is then followed by transcription regulatory events that lead to a prolonged reorganization of cellular status [39]. The cellular environment is subject to continuous and unpredictable changes. To deal with such changing conditions, biological systems require robustness, a ubiquitous property that allows cells to maintain their central functions in the face of external or internal perturbations [39,40]. Several redundancies and compensatory mechanisms provide the system with sufficient flexibility in response to various stimuli, and support it to overcome even the most severe attacks without becoming fragile.

Systemic chronic diseases can be seen as a manifestation of co-opted robustness, in which normal physiological mechanisms are efficiently taken over to sustain and promote an epidemic, and potentially a more progressive disease status [40]. An optimal drug should render the system fragile by performing perturbations for which the system has not been optimized. Thus, the probability of a system breakdown should correlate with the number and diversity of the target-affecting agents applied [41-43].

## Discussion

### The enormous complexity of multicomponents is a challenge for activity monitoring

Elucidating the risks and benefits of multicomponents, in particular, with respect to their efficacy and safety, is a prerequisite for their (re-)entry into standard therapies. These requirements are not achievable via conventional drug assessment strategies, since the plethora of multicomponent-mediated effects does not make it feasible to accomplish this via reductionist approaches.

Predictions of combinatorial effects of reference listed drugs are typically extrapolated from classical monosubstance toxicity data. This approach was applied, for example, by Borisy *et al.*, who discovered new activities using a systematic screening method for identifying effective two-component drug combinations [9]. In some cases, the molecular basis of newly emerged combined effects could even be unravelled. However, such descriptive theoretical approaches cannot be applied to a combination of multiple compounds [15,28].

Botanical and natural product multicomponents, that are integral parts of traditional medicine, have been discovered mostly by serendipity and have been developed and adapted to contemporary requirements over generations [6]. To-date, the best confirmations of their therapeutic efficacy are from their practical application. In contrast to constituted synthetic combinations, many traditional plant extracts or polyherbal remedies contain not only substances that are responsible for targeting the disease, but also components that are responsible for reducing adverse effects and/or contribute only indirectly to the net effect (e.g. by enhancing bioavailability). Unfortunately, some of the active ingredients of botanical multicomponents cannot be characterized, and thereby make it impossible to exactly identify each and every individual component. The multicomponent concept of effect potentiation, as well as low doses of "principle" constituents, is hardly feasible with the currently available chemical and molecular biological approaches. Therefore, an implementation of new strategies to understand joint effects should be emphasized. This demand is also supported by other multi-disciplinary areas, such as nutrition or ecotoxicology sciences [44].

### Multiple drug actions complicate risk-benefit assessment strategies

The pharmacokinetics and pharmacodynamics of a drug are usually evaluated by addressing toxicology and efficacy of the main active compound(s) rather than by taking into account the combinatorial effects. Most strategies for drug activity evaluation are hypothesis-driven and focus on confirming the primary function of an active substance, which is frequently predicted by structural comparative analysis. Furthermore, the obtainable information from traditional toxicological assays is limited due to their inability to recognize latent toxicity.

Interdisciplinary approaches, which combine pathophysiological and genetic information with biochemical and cell biological assays, are increasingly being used to study drug effects on one or more genes or proteins in a single pathway [45]. However, these approaches are still unsatisfactory in characterizing the multitude of responses that occur in a perturbed system [46].

Furthermore, dosage and dose range, drug interactions, and safety studies are much more complex for mixtures [4]. Often, the requirement for additional data on toxicology, mechanism(s) of action, pharmacokinetics, and drug-drug interactions, which would be necessary for the commercialization of a multicomponent as a drug, cannot be accomplished using conventional methodologies in an economical way [3]. Due to a more favourable regulatory environment, many herbal remedies are commercialized as nutraceuticals or dietary supplements, although the biological activity may be of pharmacological relevance and goes far beyond the supply of nutrients, vitamins or minerals [12].

Activity monitoring of multicomponent drugs requires a reconsideration of the conventional methods, and an implementation of new strategies that allow for an integrated overview of the participating molecular processes, which in combination mediate activity and effectiveness. Since in living systems molecules act in a non-linear and concerted manner rather than isolated, it is necessary to focus on multiple genes, proteins, and metabolites [47]. Thus, the main activities of a complex mixture have to be deduced from the functional changes of a system in an unbiased and global manner. The integration of gene expression signatures and phenotypic endpoints from *in vivo *data, in addition to chemical and biochemical information, is also gaining importance in drug research on monosubstances [5,48].

### Network architecture and biological processes

Network biology gives further insight into the importance of a multiple target approach to override compensatory mechanisms [5]. Furthermore, it supports the importance of focusing on the actions of drugs on cellular processes and biological functions rather than on a single molecule in a pathway. New approaches that include the use of large datasets on a transcript-, protein-, or metabolite-level have already started to become part of the drug assessment process, where the main goals are to define toxicological mechanisms, identify biomarkers, and use expression signatures for predicting drug effects.

Networks models, for example those that build up the cellular interactome, are novel initiatives that aid in visualizing the hierarchical structure of highly interconnected biological functions. Such models can be generated from any type of large-scale datasets, and are usually completed with building information that can be derived from several interaction databases. A network is composed of subnetworks or modules. Network cores are built up of highly conserved and robust parts that are connected with input and output subnetworks to provide feedback and control loops. The connections between the various components within a subnetwork, as well as the inter-subnetwork connections, have to balance between being too populated to enable information flow and too sparse to counteract rapid genome-wide effects [39]. Additionally, there is a functional redundancy of certain components of a system to maintain its stability. That is, in case of inactivation, a molecule's function can be compensated for by another molecule. Furthermore, the modular architecture of cellular networks enables them to restrict perturbations to a certain locale, and thereby protect the global system. In general, the robustness of a system can be affected by the removal of network nodes or by sustaining malfunctions [29].

Multicomponent therapeutics may be advantageous as they likely operate through multiple weak perturbations. These signals may undergo parallel processing in quasi-independent subnetworks prior to resulting in an integrated effective response. Additionally, a multilateral attack on the cellular system may also circumvent or delay the development of resistance mechanisms [23,29,39,49]. These multilateral perturbations can be seen on both a cellular- and an organism-level. In some diseases, the contemporary perturbation of many dysfunctional cell types, which are distributed throughout the body, could help to control disease progression in a systemic manner.

### Network-based analysis of gene expression data in multicomponent activity monitoring

High throughput screenings (HTS) using omics- (e.g. transcriptomics, proteomics and metabolomics) and functional genomic technologies are bio-analytical approaches for detecting global molecular changes in a cell system upon exposures to any kind of perturbation. HTS data analysis can be used as a pragmatic approach to systematically evaluate the activity of mono- and multicomponent drugs, and study combinatorial effects at a molecular level [28,47]. In the recent past, there have been attempts to utilize gene expression analysis to define toxicological endpoints and to elucidate mechanisms of toxicity [49]. However, global technologies, including cDNA microarrays, mass spectrometry, or protein chip data, which may provide predictive gene signatures of toxicity or carcinogenicity, proceed slowly due to the high costs associated with such experiments, the large amount of data that is generated, and the difficulty of interpreting these data in an adequate manner [18]. Additionally, a lack in standardization and validation experience is a hindrance for employing such alternative test methods in regulatory decision making processes.

The starting point for the acquisition of global sets of biological data may emanate from different levels of analysis, starting from DNA or RNA to protein or metabolites. In many cases, microarrays are used as a standard tool for studying global gene expression patterns in response to a changed environment. Since transcription, as the first step in gene regulation, is required for the dynamic adaptation of the cells proteome to different demands, a change at the transcriptional level would also deliver information regarding their associated biological processes [50,51]. However, information from gene expression data is limited, and not all drug-induced alterations can be monitored. Protective mechanisms have to be initiated at the instance of a perturbation, and therefore, have to be quicker than the biological processes, which require active biosynthesis. Such changes may include protein modification and redistribution, or changes in intra- or extracellular metabolite levels, and can be visualized only via proteomic or metabolomic approaches. However, these processes are usually followed by regulatory events at the transcription level [28,51]. Long-lasting regulatory cascades are rare in sensory and nutrient responses, as they need to react robustly and rapidly against external signals. It has been shown that a limited set of regulation patterns, which carry out specific information-processing functions to control gene expression spatially and temporally, occur repeatedly throughout a network [51,52]. Thus, analyzing the reorganization of gene expression signatures may provide insight into initiated cellular programs, as well as into the metabolic status of the cell.

To realize the full potential of global technologies in risk-benefit assessments, the integration of expression profiles with multiple data resources (e.g. conventional toxicity data, toxico-and pharmacogenomic profiles, or physical interaction maps available within public databases), as well as iterative biological modelling is required [18,53]. A compilation of bio-molecular network structures would be helpful in reorganizing a vast collection of data, and making them more accessible and valuable with respect to their information on cellular functions and processes.

### Analysing gene expression data

**Figure 1:**
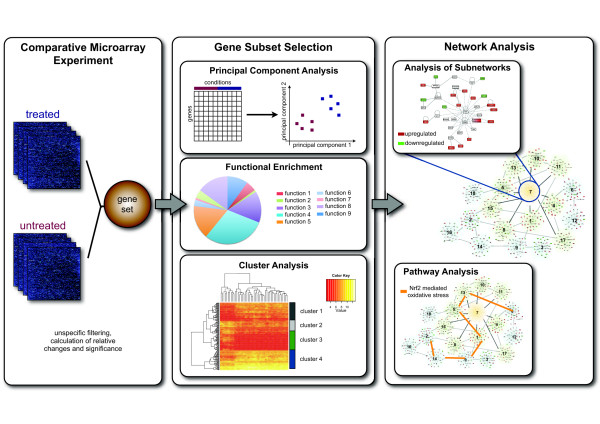
**Example of a comparative microarray experiment (treated versus untreated or control)**. The resulting intensity data are filtered non-specifically to reduce the number of hypotheses to be tested. In the next step, relative expression values and p-values are calculated for every single gene. After geneset reduction, several sophisticated methods for gene subset selection can be applied. Principal component analysis (PCA) is mainly used for dimensionality reduction. PCA enables the visualization of multidimensional datasets and can be effectively applied for gene selection. Other approaches for subset selection are functional enrichment strategies. These methods result in sets of aggregating genes with similar functions. Cluster analysis can be used to group genes according to their expression similarity. Clusters of genes with similar expression profiles can be used as starting points for further bioinformatic analyses. Network analysis is a method where an interaction network is constructed by integrating the geneset with direct or indirect molecular relationships extracted from various biological knowledge bases. The subnetworks show a distinct degree of interconnectivity. For example, subnetwork 7 appears to contain interactions that are responsible for some important process or behaviour, as its removal would affect the entire network. (Herein, network images were created via Ingenuity^® ^Pathway Analysis (IPA); http://www.ingenuity.com).

An extraction of bio-molecular subsets that are determinants of cellular status from large datasets remains a major challenge in the interpretation of HTS data. Similar to other types of data, there are different strategies available to uncover groups and patterns of co-regulation or causal relationships. Herein, several important secondary data analysis strategies for uncovering conserved patterns from highly dimensional gene expression data are briefly presented along with their advantages and limitations (Figure 1).

Sorting target molecules according to expression levels (relative to control) is the fastest and easiest way of analysis, but does not deliver much mechanistic information. Differential expression of gene sets cannot discriminate between directly regulated and secondary targets. Since genes that belong to the same complex or regulatory pathway tend to have correlated expression profiles, an identification of patterns and expression classes provides a much better insight into their biological functions [51].

#### Principal component analysis (PCA)

Principal component analysis (PCA) is a method for reducing high dimensional data spaces into lower dimensional ones, while retaining most of the variation in the dataset. Lower dimensional data spaces are easier to visualize and interpret. Similarities and differences between samples can be visualized by plotting. Since standard PCA is based on a linear dimensionality reduction, modern variations of PCA are using non-linear curves to overcome the limitations of linearity [54,55].

#### Clustering ("unsupervised learning") and classification ("supervised learning")

Clustering methods result in sets of genes that are associated with a particular cellular state. Multivariate clustering techniques calculate a measure of similarity between gene expression profiles and are used for unsupervised analysis of gene expression data (i.e. hierarchical clustering, K-means clustering, self-organizing maps) [56-59]. Supervised analysis strategies are used to assign unknown members into known groups by using, for example, linear discriminants, decision trees or neural networks, while two-way clustering methods are used to discover genes that are co-regulated only within a subset of experiments [60].

Given the high number of clustering and classification algorithms, it remains difficult to select an appropriate algorithm based on distance measures, linkage rules and many other parameters. Furthermore, the causality of gene co-expression, and the functional relationships between clusters are not explored, which makes it challenging to elucidate biological mechanisms [47,51]. Nevertheless, cluster information can be an effective basis for further analyses based on *a priori *functional knowledge.

#### Functional enrichment strategies and pathway analysis

To address the issue of causality in shared expression patterns, biological knowledge accumulated in public databases, such as Gene Ontology, can be used to dissect genes that are involved in a specific biological process, molecular function, or cellular component [61]. Many other similar tools are publicly available, and can contribute to the functional analysis of larger gene sets (i.e. Onto-Express, MAPPFinder, GOMiner, DAVID, GeneMerge, FuncAssociate) [62-68]. Huang *et al. *classified bioinformatic enrichment tools according to their underlying algorithms [62]. However, the development of precise guidelines for choosing the most appropriate enrichment tool is likely impossible, as research projects differ in their needs and the questions being asked.

Mapping expression data into precompiled pathways is one way of obtaining direct biological or toxicological relevant information. However, it is important to keep in mind that the activity of a pathway depends not only on the abundance, but also the activity of its components. Gene expression data may reflect mRNA abundance, while proteins are further regulated through turnover rate, activity, posttranslational mechanisms, and interactions [69]. Furthermore, redundant and divergent mechanisms contribute to a pathway's activity [15].

A limitation of functional enrichment strategies is that they are restricted to annotated information that is stored in the database and may be subject to change. Although these methods enable the identification of coherent expression changes, the discovery of new pathways of organizational units, which have no records in the database, is not possible [47].

#### Network analysis

A better understanding of molecular relationships must be obtained via the building, validation, and analysis of mathematical models to gain insight into cellular processes or even predict cellular behaviour from transcriptional signatures [70]. The construction of gene network architectures from expression profiles is also often referred to as "reverse engineering" [51]. A network topology cannot be easily derived from literature on precompiled pathways, as networks are typically not static and change within context and time, as well as because cells constantly adapt to their internal conditions in response to internal and external stimuli [71]. The construction of a network relies on information regarding direct or indirect molecular relationships. Aside from the topology of connections, different mathematical models and network constructing algorithms can also include information on the causality from the directionality of relationships, information on the type of effect (i.e. activating, stimulatory, inhibitory), the strength of the interaction, and, in some few models, the kinetics and dynamics. For the last two points, a high and often unpredictable number of experiments are required to temporally resolve and untangle individual interaction wires, as well as reconstruct the regulatory strengths of each component in relation to others [70].

Network approaches, however, do allow for the integration of diverse types of data in the construction of a biological model. Such a model may reveal important, but not apparent, relationships, as well as identify which non-differentially expressed molecules are actually regulatory molecules [51]. Furthermore, it is insufficient to map only physical components and interactions for assessing biological functions. It is also necessary to evaluate how information propagates through a system. A broad range of information is available from an integration of gene expression data and previous biological findings, such as gene ontology (GO) categories or findings from different cell types, organs, and even organisms. However, this again depends on what scientific question is being addressed, and what restrictions are being imposed to get the most relevant biological information out of the network.

The limitations of both functional enrichment and network analysis are the quality and comprehensiveness of databases available, including the nature of the stored information (e.g. limited knowledge, interaction discovered mainly *in vitro*), as well as their algorithms, which do not or only very limited allow for dynamic network modelling. Therefore, highly accurate mathematical models for predicting network behaviour are only applicable for certain situations.

## Conclusion and perspectives

Every therapeutic intervention, either a mono- or multicomponent drug, results in changes of intra- and intercellular signalling events, and finally leads to pleiotropic effects that affect an organisms' homeostasis. In particular, concerning multicomponent activity monitoring, new concepts that overcome the limitations of conventional risk-benefit assessment strategies are urgently warranted.

**Figure 2:**
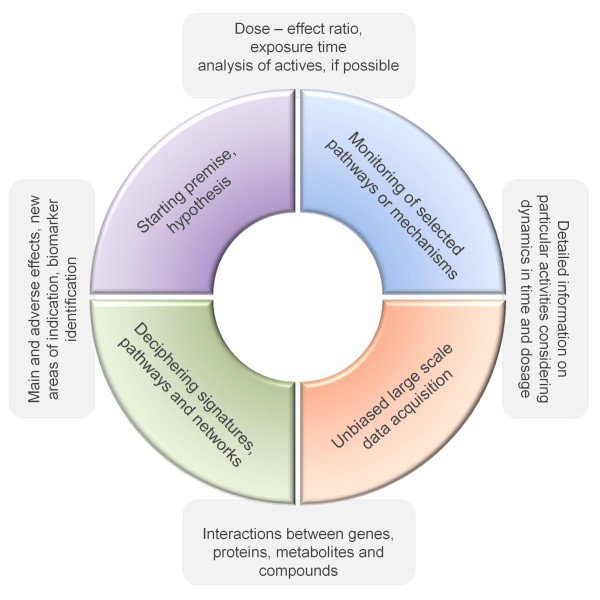
**An iterative strategy for comprehensive activity monitoring and risk-benefit assessments in multicomponent drug research**.

Taking into consideration the main and adverse effects, a strategy that combines both selected pathway-or interaction-based bioassays and unbiased analysis of expression signatures, would be useful to fully assess the pharmacological properties of a multicomponent. As shown in Figure 2, in general, some information on the proposed effects of a multicomponent is required to generate hypotheses and design preliminary experiments. This information can be either retrieved from traditional application areas or deduced from the multicomponent's ingredients. Consequently, more detailed dose-effect relationships can be further analysed via assays that focus on particular activities, and aid investigators in extracting the necessary parameters for assessing large-scale quantitative data. For instance, a respectable number of versatile and reliable bio- and reporter gene assays are available for elucidating the transcriptional activities of particular signalling pathways [45].

Unbiased large-scale data acquisition strategies give insight into transcriptomic, proteomic, and/or metabolomic alterations, which provide more information on all of the activated molecular processes in a system. The integration of such datasets with comprehensive knowledge bases containing direct and indirect molecular interactions aids in deciphering the most prominent modulated pathways. Furthermore, network analysis is a useful tool for reorganizing a vast collection of data in a way that affected functional modules in a cellular network and biological information flow throughout the system can be visualized. Also, key molecules or potential biomarkers can be identified within a network. Finally, the evaluation of omic data may refine the experimental model in order to make better predictions, which will then be tested with new experiments [72]. Hence, the extraction of valuable information from large datasets for multicomponent activity assessments requires an iterative approach.

Aside from deciphering the main mechanisms of action, if some of its components interfere with unexpected processes, side effects of the remedy can be also detected. Furthermore, low dose extrapolations are supported and additional therapeutic opportunities or effects specific to certain populations may be uncovered [15,18,29]. To-date, transcriptional profiling is the most frequently used large-scale data acquisition methodology in clinical research. Currently, microarray technology is less expensive and even more developed, in comparison to other techniques applied in proteomic and metabolomic research. However, it should be kept in mind that mRNA levels detected on microarrays are steady-state abundance levels, which also depend on transcription and degradation rates [69]. Furthermore, direct and indirect effects cannot be distinguished, and in some cases, these discrepancies may interfere with the deduced biological information.

To avoid pitfalls in applying omics approaches to multicomponent activity analysis, for example, by generating datasets that are too large to deal with or by choosing inadequate parameters, a rigorous experimental design is necessary. The application of fractional factorial designs and statistical methods, which consider the need for replicating experiments and resources, may reduce experimental efforts [40,73,74]. However, there is still need for improvements in experimental design that capture the multivariate nature inherent to biological regulatory networks. At the moment, this can be addressed only through the use of predictive mathematical models [75].

Thus, the application of large-scale data acquisition technologies is limited, as they consume more time and financial resources in comparison to biased assays. In addition, even the largest experimental setup would not provide sufficient information to construct a fully detailed mathematical model with high statistical confidence. Consequently, data interpretation is not always straightforward and conclusive [51]. However, global systemic approaches are highly recommended to generate new hypotheses, and assist in the selection of potential biomarkers and additional focused analysis strategies.

Selecting an appropriate cellular model system may also have a great impact on the results. For example, primary materials, such as donor blood cells, may provide a more *in vivo*-like situation regarding sensitivity and behaviour than artificially immortalized cell lines. Additionally, models that mimic a particular disease, either via a genetic or chemically-induced manipulation, could provide more relevant information on a specific question. However, cellular model systems are limited in that they cannot truly imitate an entire organism, where the response to external stimuli is regulated on several hierarchical levels (e.g. sensory organs, nervous signal transduction system, organ systems, tissue, etc.). Thus, knowledge regarding cellular targets, as well as their robustness and fragility in disease onset and progression, remains a fundamental factor that determines also the success of new system-based strategies [28].

It should be noted that also nutritional sciences benefit from multimixture analysis approaches. Metabolic pathways and homeostasis are disturbed in many diet-related diseases [13]. Additionally, beneficial drug-food interactions may contribute to the therapeutic successes of drugs not only by improving the patient's general condition, but also by reducing side-effects [76].

Omics technologies for multicomponent activity assessments cannot only be applied for endpoint analysis in mammalian systems, but can also be useful in plant phenotyping and extract standardization. In addition to improving phytochemical identification by coupling bioassays to fractionation steps, metabolomic methods are being more and more applied, as they allow for the study of thousands of secondary metabolites in a complex mixture, without the need for isolating active principles. The chemical profile of a preparation can then be linked to observations obtained through biological testing systems [77].

Hence, the combination of both biased and non-biased assays appears to be the most promising strategy for not only risk-benefit assessments, but also drug design, identifying drug targets, and biomarkers. This concept may aid in deciphering condition-specific regulations of a system in response to a dynamic environment, as well as contribute to the general understanding of the interactions between genes, proteins, metabolites, nutrients, drugs, and environmental factors in healthy and diseased states.

## Competing interests

The authors declare that they have no competing interests.

## Authors' contributions

All authors discussed the topic. JMG drafted the manuscript, MJ and OAW contributed to the conception, FÜ and DF critically revised the manuscript. All authors read and approved the final manuscript.

## Pre-publication history

The pre-publication history for this paper can be accessed here:

http://www.biomedcentral.com/1472-6882/12/18/prepub
